# Genome Sequences of Soft Rot-Causing Pseudomonas Isolates from Spinach

**DOI:** 10.1128/mra.00701-22

**Published:** 2022-08-22

**Authors:** Chloe Wasendorf, Dylan L. Schultz, Stephan Schmitz-Esser, Nick T. Peters

**Affiliations:** a Interdepartmental Microbiology Graduate Program, Iowa State University, Ames, Iowa, USA; b Department of Animal Science, Iowa State University, Ames, Iowa, USA; c Department of Plant Pathology and Microbiology, Iowa State University, Ames, Iowa, USA; University of Maryland School of Medicine

## Abstract

Two Pseudomonas strains (SR17 and SR18) were isolated from soft rot-diseased spinach leaves. Here, we report their genome sequences and characteristics.

## ANNOUNCEMENT

Soft rot disease of plants, which is a deterioration of plant tissues resulting in smelly, mushy, and inedible fruits and vegetables, can be caused by many pathogens. *Pectobacterium* and *Dickeya* species represent the leading disease-causing agents in the field and postharvest; however, other bacteria, like Pseudomonas spp., contribute to disease instances as well (1–7). Soft rot plant pathogens secrete plant cell wall-degrading enzymes, including pectin lyases, proteases, and cellulases, to macerate plant cells and tissues (8). Once disease symptoms occur, treatment is impossible, so prevention of this disease is key. We have isolated two soft rot-causing bacteria from spinach, both of which were Pseudomonas strains, SR17 and SR18; here, we provide their draft genomes.

**FIG 1:**
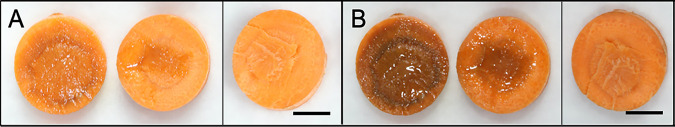
Examples of soft rot caused by the sequenced Pseudomonas isolates on carrots. Carrots were inoculated with 10 μL of an overnight culture of (left to right in each panel) SR17, SR18, or a sterile buffer, with SR17 and SR18 incubated together at 25°C in a moist chamber and the control incubated separately at 25°C in a moist chamber. Pictures were taken at 48 (A) and 72 (B) hours after inoculation. Dark, wet, and mushy spots are symptoms of soft rot. Bar, 1 cm.

The soft rot phenotype was confirmed by swabbing rotted material from spinach, gathered from grocery stores (Ames, IA, USA) in 2019, onto sterilized carrot slices (9) and after incubation at 25° in a moist chamber for 48 h, isolating the bacterial communities from the diseased carrots on Luria-Bertani (LB) agar. Pure cultures from the LB plates were then individually swabbed onto sterilized carrot slices and incubated as before to confirm the soft rot phenotype. Examples of soft rot caused by these isolates on carrots are shown in Fig. 1. Sanger sequencing of the 16S rRNA gene PCR products using common 16S rRNA primers, 27F (5′-AGAGTTTGATCMTGGCTCAG-3′) and 1492R (5′-GTTACCTTGTTACGACTT-3′), for amplification and a search conducted using NCBI BLASTn against the NCBI nonredundant (nr) database identified the isolates as Pseudomonas species (10). For genome sequencing, DNA extraction was performed using the Nanobind CBB Big DNA kit (Circulomics, Baltimore, MD). Sequencing was conducted using Illumina MiSeq 250-bp read length paired-end sequencing at the Iowa State University DNA Facility, and 603.3 Mbp of total sequence data was generated across 1.25 million and 1.16 million reads for SR17 and SR18, respectively (Table 1). Library preparation was performed using the NEBNext Ultra II FS kit with standard parameters. FastQC v0.11.9 was used to assess the read quality (note: default settings were used for all software unless specified otherwise) (11). Bases below a quality score of 20 were trimmed and adapter sequences were removed using BBDuk v37.36 with the following options: “ref=adapters.fasta ktrim=r ordered k=23 hdist=1 mink=11 tpe tbo qtrim=w trimq=20 minlen=75” (12). Only trimmed reads longer than 75 bp were used to generate genome assemblies with SPAdes v3.14.1, using the “–careful” option (13). Annotation of the assembled genomes was performed using the PATRIC database and the NCBI PGAP (14, 15).

**TABLE 1:** Pseudomonas isolates and genome characteristics

Isolate	Taxonomy	Assembly size (Mbp)	No. of contigs	GC content (%)	Source	GenBank accession no.	Raw read data (SRA accession no.)	No. of raw Illumina reads	Total sequence data (Mbp)	Contig *N*_50_ (bp)	Coverage (×)
SR17	Pseudomonas sp.	6.83	48	60.3	Spinach	JAMQAV000000000	SRR19546780	1,252,410	313.1	274,304	34
SR18	Pseudomonas sp.	5.97	48	60.5	Spinach	JAMQAU000000000	SRR19546779	1,160,792	290.2	255,521	34

The genome sizes ranged from 5.97 to 6.83 Mbp, had 48 contigs each, and had a GC content of approximately 60% (Table 1). Average nucleotide identity (ANI) results showed that SR17 and SR18 are 88.9% similar to each other. SR17 is closely related to Pseudomonas marginalis ICMP 3553 (GenBank accession number GCA_001645105.1; ANI, 98.9% with 90.4% overlap) and Pseudomonas fluorescens SBW25 (GCA_000009225.1; ANI, 98.4% with 87.2% overlap), while SR18 is closely related to Pseudomonas cyclaminis MAFF 301449 (GCA_015163715.1; ANI, 96.1% with 79.2% overlap). This work provides the basis for further experiments into these agriculturally and economically important plant pathogens.

### Data availability.

The Pseudomonas sequences have been deposited at GenBank under the BioProject accession number PRJNA844386. GenBank and SRA accession numbers for each isolate are listed in Table 1.
